# Machine Learning for Data Linkage

**DOI:** 10.23889/ijpds.v8i2.2242

**Published:** 2023-09-14

**Authors:** Lucy Robinson

**Affiliations:** 1 Health inequalities in Covid-19 deaths and hospital admissions in Wales

## Objectives

This analysis examines the impact of confounding/mediating factors on health outcomes, comparing inequalities in COVID-19 associated hospitalisations and deaths with inequalities in other causes of admissions. This includes how socioeconomic inequalities are explained by mediating factors such as underlying health conditions, ethnicity, and occupation.

## Method

We created a cohort of the Welsh population using anonymised individual-level linked data in the Secure Anonymised Information Linkage (SAIL) Databank. We used logistic regression of COVID-19 outcomes using the Welsh Index of Multiple Deprivation (WIMD) as a predictor to understand how the coefficient for WIMD is changed when other variables (such as comorbidities, smoking status, BMI, etc.) are added. Other variables including overcrowded housing and occupation, were also explored to further understand whether these kinds of confounding factors may mediate inequalities or if outcomes are more influenced by other unobserved causes.

## Results

Initial analysis shows that age-standardised hospital admission, ICU and death rates were roughly twice as high in the most deprived vs least deprived WIMD quintiles in Wales. The overall slope index of inequality was estimated to be 215, meaning the gradient of mortality is 215 per 100,000 higher in the most deprived WIMD quintile compared with the least deprived WIMD quintile. Thus, showing an inequality gap in relation to COVID-19 deaths between the most and least deprived. We estimated Years of Life Lost per 1,000 due to COVID-19 as 28.9, with a variation between WIMD quintiles of 38.4 for the most deprived and 22.2 for the least deprived. The project produced regression formulas to see the contribution of different confounding factors.

## Conclusion

This study built on existing research findings and further explored the reasons for known socioeconomic inequalities in relation to COVID-19. Insights from this study have broadened the knowledge around the impact of mediating factors and could therefore contribute to future pandemic preparedness.

